# Structural changes in troponin during activation of skeletal and heart muscle determined in situ by polarised fluorescence

**DOI:** 10.1007/s12551-024-01245-y

**Published:** 2024-10-19

**Authors:** Ivanka R Sevrieva, Thomas Kampourakis, Malcolm Irving

**Affiliations:** 1 Randall Centre for Cell & Molecular Biophysics, New Hunt’s House, Guy’s Campus, King’s College London, London, UK; 2 Division of Cardiovascular Medicine, College of Medicine, University of Kentucky, Lexington, KY USA

**Keywords:** Troponin, Skeletal muscle, Heart, Calcium, Muscle regulation

## Abstract

Calcium binding to troponin triggers the contraction of skeletal and heart muscle through structural changes in the thin filaments that allow myosin motors from the thick filaments to bind to actin and drive filament sliding. Here, we review studies in which those changes were determined in demembranated fibres of skeletal and heart muscle using fluorescence for in situ structure (FISS), which determines domain orientations using polarised fluorescence from bifunctional rhodamine attached to cysteine pairs in the target domain. We describe the changes in the orientations of the N-terminal lobe of troponin C (TnC_N_) and the troponin IT arm in skeletal and cardiac muscle cells associated with contraction and compare the orientations with those determined in isolated cardiac thin filaments by cryo-electron microscopy. We show that the orientations of the IT arm determined by the two approaches are essentially the same and that this region acts as an almost rigid scaffold for regulatory changes in the more mobile regions of troponin. However, the TnC_N_ orientations determined by the two methods are clearly distinct in both low- and high-calcium conditions. We discuss the implications of these results for the role of TnC_N_ in mediating the multiple signalling pathways acting through troponin in heart muscle cells and the general advantages and limitations of FISS and cryo-EM for determining protein domain orientations in cells and multiprotein complexes.

## Introduction

**Fig. 1:**
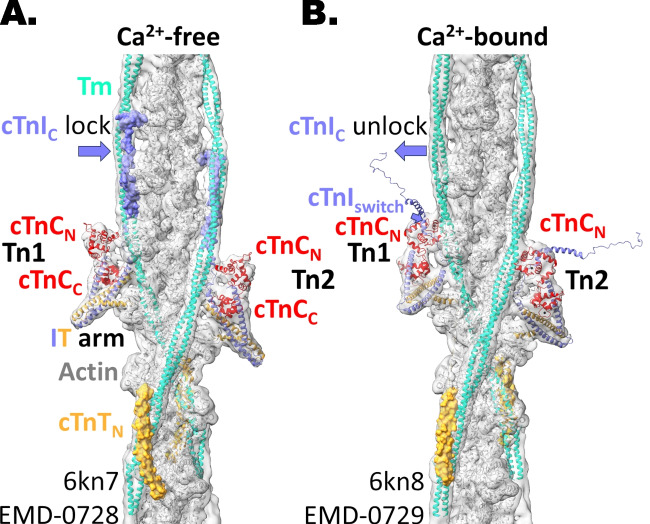
The cardiac thin filament in the Ca^2+^-free or APO state (**A**) and the Ca^2+^-bound state (**B**), from Yamada et al. 2020 (PDB 6kn7 and 6kn8, respectively) fitted into the cryo-EM density volumes (EMDB accession codes EMD-0728 and EMD-0729, respectively). Actin is oriented with its pointed end up. The two troponins (Tn1 and Tn2) are non-equivalent and elongated, spanning seven actin monomers and cross-linking tropomyosin (Tm) molecules on opposite actin strands. The N-terminus of troponin T (TnT) crosses over the head-to-tail junction of Tm, which is located farther away from the C-terminal domain of troponin C (TnC_C_) by one actin subunit in Tn1 as compared to Tn2. In the APO state, the C-terminus of troponin I (TnI_C_) lies parallel to and binds tropomyosin (Tm) and actin covering myosin binding sites (TnI_C_ lock). In the Ca^2+^-loaded state, the N-terminal domain of troponin C (TnC_N_) binds a helical region of TnI_150-163_ or switch region (TnI_switch_), which fits into the exposed hydrophobic pocket in TnC_N_, thus lifting TnI_C_ from the thin filament (TnI_C_ unlock). A model of TnI_160-210_ is shown for illustration purposes only as the density for TnI_167-210_ is absent in the Ca^2+^-bound state. The crystal structure of TnC_N_ cannot be fitted reliably in either the Ca^2+^-free or Ca^2+^-bound state due to the lower resolution of the cryo-EM density maps in that region and the globular shape of the N-lobe. The colour code is actin (grey), Tm (bright mint), TnT (bright orange), TnI (slate) and troponin C TnC (red)

Contraction of skeletal and heart muscle is triggered by calcium binding to troponin in the actin-containing thin filaments (Gordon et al. 2000). In resting skeletal muscle and in heart muscle between beats (in diastole), when the intracellular calcium concentration [Ca^2+^]_i_ is about 50 nM, troponin and its partner regulatory protein tropomyosin bind to actin to block the binding of myosin motors (Fig. 1A), preventing the interaction between myosin, actin and ATP that drives contraction. Depolarisation of the muscle cell membrane triggers a transient increase in [Ca^2+^]_i_ to about 1 µM in heart muscle and about 10 µM in skeletal muscle, allowing Ca^2+^ to bind to the regulatory site on troponin in heart muscle and the two sites on troponin in skeletal muscle. This leads to a change in the conformation of troponin and tropomyosin on the thin filaments that uncovers the myosin binding sites on actin (Fig. 1B), allowing myosin motors to bind to the thin filaments and drive filament sliding and force generation.

The basic concept of this ‘steric blocking’ model of muscle regulation was proposed more than 50 years ago (Huxley 1972; Parry and Squire 1973), soon after troponin was identified as the protein component that conferred calcium sensitivity on the contractile system (Ebashi et al. 1969). Nevertheless, and despite recent dramatic progress in understanding thin filament structures driven by improvements in cryo-EM technology (Oda et al. 2020; Yamada et al. 2020; Risi et al. 2021b, 2024), many aspects of the molecular structural basis of thin filament regulation remain obscure (Tobacman 2021). This is partly due to the complexity of the thin filament structure (Fig. 1), in which the fundamental repeating unit contains 14 actin monomers, two tropomyosins and two troponins. The actin monomers are arranged in a two-stranded helix. Tropomyosin, a long α-helical coiled coil consisting of seven homologous actin-binding repeats, follows the strands of the actin helix (von der Ecken et al. 2015; Yamada et al. 2020) (Fig. 1). However, troponin does not follow the helical symmetry of actin, and the two troponins in the repeating unit of the thin filament are not identical. Moreover, whereas two regions of the so-called core domain of troponin have well-defined secondary and tertiary structure that has been described at atomic resolution by crystallography and NMR, most other regions are dynamically disordered, and some of them have not been resolved in electron density maps determined by cryo-EM. To be consistent in comparisons between different structures, we will refer to residue numbers including the initial methionine.

The simplest of the two well-defined regions of the core domain of troponin is the N-terminal lobe of the calcium-binding subunit of troponin, which we will refer to as TnC_N_. This region is also called the ‘regulatory head’ of troponin (Takeda et al. 2003) because it contains the regulatory Ca^2+^ binding sites, of which there are two in the skeletal muscle isoform and one in the cardiac isoform. The Ca^2+^-bound form of TnC_N_ also binds a short sequence of the I chain of troponin (TnI) called the switch region (Fig. 1B), and this binding step is likely to be important in the regulatory mechanism, as discussed below. The other well-defined region of the troponin core domain, called the IT arm (Takeda et al. 2003), contains parts of troponin T (TnT), TnI and the C-terminal lobe of TnC (TnC_C_), with the latter clasped tightly between long, almost parallel helices of TnI and TnT.

This review focuses on the orientation of the regulatory head and IT arm of troponin in thin filaments and on the functional implications of the changes in orientation of those two domains for the regulation of muscle contraction. Those orientations are likely to be different in isolated thin filaments and in muscle cells, for several reasons. First, the regulatory state of the thin filament is altered by myosin binding (Gordon et al. 2000; Brunello et al. 2023). The calcium dependence of the ATPase activity of isolated myosin motor domains in the presence of reconstituted thin filaments led to a model in which the thin filament is considered to have three regulatory states called *blocked* (in the absence of both Ca^2+^ and myosin), *closed* (with Ca^2+^ but not myosin bound) and *open* (with both Ca^2+^ and myosin bound) (McKillop and Geeves 1993). These three states have been correlated with distinct azimuthal positions of tropomyosin, using rigor (ATP-free) conditions as a model for the open state (Lehman et al. 1994). The extent to which these three in vitro states are populated during contraction in physiological conditions is considered further below on the basis of changes in the orientation of the regulatory head and IT arm in muscle cells.

Second, thin filament structure may be modulated by the binding of another component of the myosin-containing thick filaments, myosin binding protein-C (MyBP-C) (Pfuhl and Gautel 2012; van Dijk et al. 2014; Kampourakis et al. 2014). MyBP-C is composed of a string of Ig-like and fibronectin-like domains. The C-terminal domains are strongly bound to the thick filaments, but isolated N-terminal fragments can bind to isolated thin filaments (Shaffer et al. 2009), displacing tropomyosin towards its open state in the absence of calcium (Mun et al. 2014; Risi et al. 2021a, 2022). The effect of native, full-length MyBP-C on thin filament structure in the intact filament lattice of relaxed muscle cells in physiological conditions remains to be determined.

Third, the calcium sensitivity of contraction depends on the length of the muscle cell or sarcomere, in a phenomenon called length-dependent activation (LDA). LDA is regarded as the cellular basis of the Frank-Starling response of the heart that links the strength of ejection to the extent of the venous filling (de Tombe et al. 2010). The molecular mechanism of LDA is unknown, but it is presumably mediated by one or more components of the thick filament, myosin, MyBP-C or titin that can interact with the thin filament. These effects could clearly not influence the structure of isolated thin filaments.

Finally, the myosin-containing thick filaments also contribute to the regulation of muscle contraction (Irving 2017; Craig and Padron 2022; Brunello et al. 2023; Brunello and Fusi 2023). In resting skeletal muscle and diastolic heart muscle, myosin motors are folded back against their tails in helical tracks on the surface of the thick filament backbone, preventing their interaction with actin (Woodhead et al. 2005; Zoghbi et al. 2008; Reconditi et al. 2011; Dutta et al. 2023; Tamborrini et al. 2023). Myosin motors are released from this inhibited state during contraction, and this transition is partly triggered by stress in the thick filament backbone (Linari et al. 2015). More generally, the regulatory states of the thick and thin filaments can be regarded as being positively coupled in an integrated dual-filament mechanism of contractile regulation (Kampourakis et al. 2016; Kampourakis et al. 2018; Kampourakis and Irving 2021; Zhang et al. 2017; Brunello et al. 2023; Brunello and Fusi 2023).

It follows from the above considerations that the full physiological repertoire of mechanisms responsible for the regulation of muscle contraction cannot be reliably extrapolated from structures of isolated thin filaments. Elucidation of the role of troponin in regulation will require molecular structural information from the intact sarcomeres of muscle cells in near-physiological conditions on the millisecond timescale of the intracellular [Ca^2+^]_i_ transient. Although such information might in principle be obtained by rapid freezing of suitable samples followed by thinning for imaging by EM, the resolution obtainable by that approach is not yet sufficient to determine the conformation of the regulatory head and IT arm of troponin in the intact sarcomere, even for samples frozen in steady-state conditions (Tamborrini et al. 2023). Some information about the azimuthal location of tropomyosin can be obtained by X-ray diffraction from intact skeletal muscle (Kress et al. 1986; Koubassova et al. 2017; Huxley et al. 1982) but the limited resolution and lack of phase information in that technique mean that it is impractical to determine the conformation of troponin domains by that method.

The focus of this review is an alternative method that can provide information about the conformation of the well-defined domains of troponin on the millisecond timescale in contracting muscle cells. Fluorescence for in situ structure (FISS) was first developed in order to determine the orientation of the light chain domain or lever arm of the myosin motor in muscle cells (Corrie et al. 1999). FISS works by replacing a target protein domain in a permeabilised muscle cell by a variant in which a pair of surface-accessible cysteine residues have been cross-linked by a bifunctional thiol-reactive fluorescent probe, thereby constraining the orientation of the probe dipole with respect to the 3D structure of the protein domain. The orientation of the fluorescence dipole with respect to the filament or muscle fibre axis can then be determined in situ from the polarisation of the fluorescence. If this approach is repeated for a set of pairs of surface residues whose relative orientations are known from structural studies on the isolated target domain, the orientation of that domain in the muscle cell can be determined.

Here, we describe the orientations of the regulatory head and IT arm of troponin determined by FISS in skeletal and heart muscle and compare them with those derived from recent cryo-EM studies of isolated thin filaments. We show that the orientation of the IT arm determined by the two methods is almost identical, within the experimental resolution, whereas that of the regulatory head is qualitatively different. We discuss the relative merits and limitations of FISS and cryo-EM in light of those results and the wider implications of the results for the role of troponin in the regulation of muscle contraction.

## Fluorescence for in situ structure (FISS)

The polarisation of fluorescence from a probe covalently attached to a target protein domain gives information about the orientation of the probe dipole with respect to the reference axes of the optical setup. If the protein is in solution, the orientations are isotropic, but it is possible to obtain information about the mobility of the probe or domain on the timescale of the fluorescence lifetime, because the polarisation is reduced if the fluorophore rotates between absorbing and emitting a photon. If the protein domain is in an oriented or anisotropic array, the polarisation gives information about the orientation of the domain with respect to the anisotropy axis. Muscle cells contain almost parallel arrays of filaments aligned with the long axis of the cell, the axis along which it generates force or shortening. Moreover, muscle cells can be considered to have cylindrical symmetry around that axis, which we will call the *z* axis. The components of muscle filaments have a defined axial orientation *β* with respect to the *z* axis, but their azimuthal orientation distribution is isotropic.

Many studies have reported polarised fluorescence from probes on muscle proteins, typically reacting an iodoacetate-conjugated fluorophore with a cysteine on a target domain. Target specificity has generally been achieved by expressing the target protein in *Escherichia coli* with a cysteine in the desired location, after replacement of any native cysteines, then exchanging the labelled protein into demembranated muscle cells. This approach works well for the regulatory light chain (RLC) of myosin and for either TnC or reconstituted troponin complex. These proteins can be exchanged for their native counterparts in relatively mild conditions in which effects on muscle function are minimal and can be characterised by control experiments (Ferguson et al. 2003; Fusi et al. 2015; Sevrieva et al. 2023). Polarised fluorescence from dipole probes attached to a single residue in a target protein can often be used to monitor changes in protein conformation in muscle cells with high sensitivity and time resolution, but the results cannot be related directly to the orientation of the target domain because the orientation of the probe dipole in the coordinate frame of the domain is unknown.

**Fig. 2:**
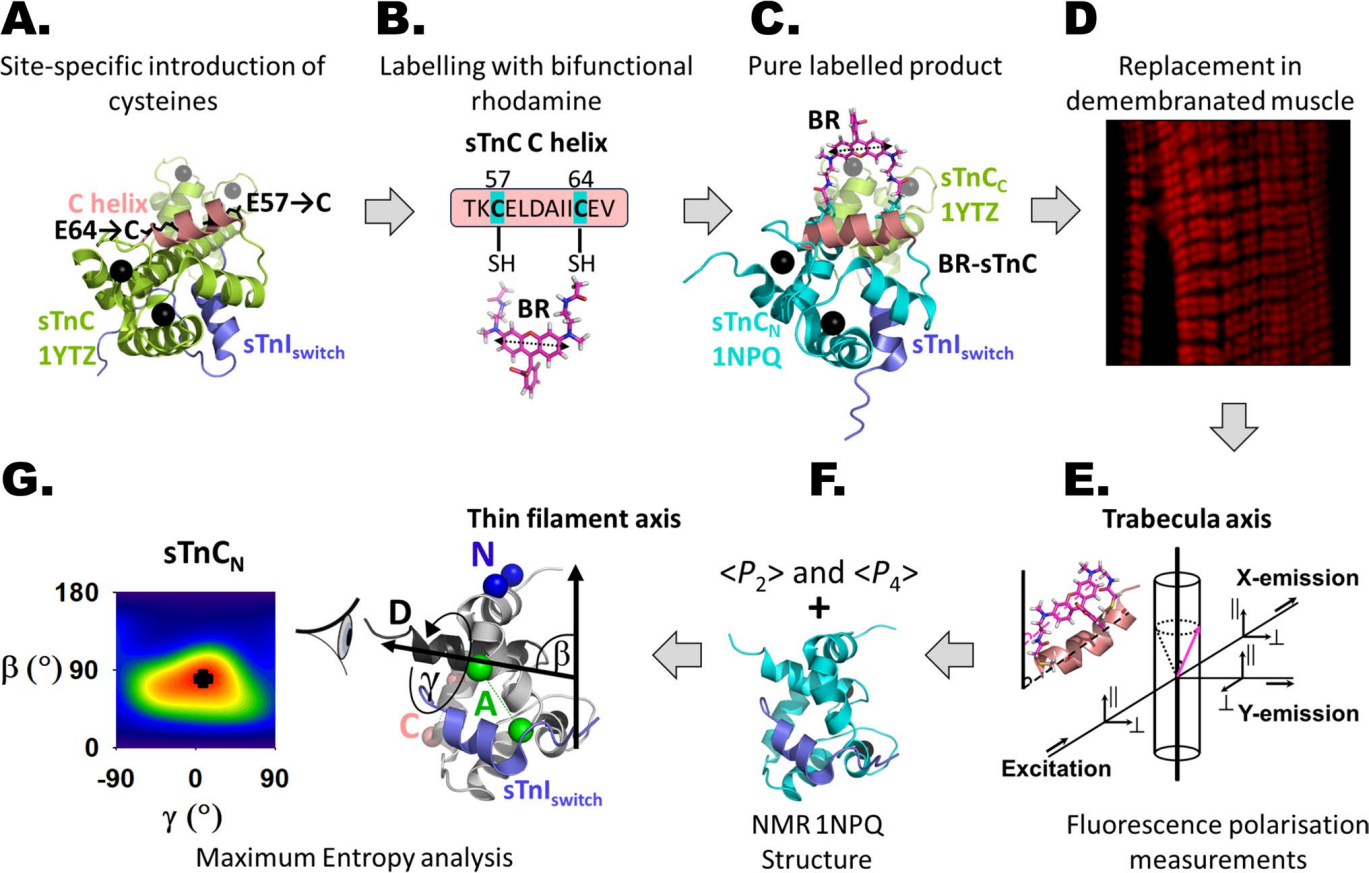
FISS workflow. **A** Cartoon representation of the calcium- and switch peptide–bound chicken fast skeletal muscle TnC (sTnC) in lemon green with C-helix coloured in salmon pink (PDB 1YTZ; Vinogradova et al. 2005). Ca^2+^ ions are shown as black spheres. The chicken fast skeletal switch peptide (sTnI_switch_) encompassing residues 115–144 is coloured in slate. Using site-directed mutagenesis, glutamic acid residues at positions 57 and 64 in sTnC (C-helix) are replaced by cysteines. **B** The recombinant double cysteine mutant TnC is expressed in *E. coli* and purified (Ferguson et al. 2003). The reactive sulphydryl groups of the cysteines in sTnC are cross-linked with a bifunctional rhodamine dye bis-iodoacetamidorhodamine (BR-I_2_), and the 1:1 BR-sTnC conjugates are purified to greater than 95% homogeneity. The double arrow indicates the fluorescence dipole, which is parallel to the long axis of the xanthene ring system. **C** Cartoon representation of the bifunctional rhodamine-labelled sTnC. The N-lobe (sTnC_N_.2Ca.^2+^.sTnI_116-132_.BR_57-64_; PDB 1NPQ; Mercier et al. 2003) is shown in cyan aligned with sTnC 1YTZ (lemon green). The BR fluorescence dipole is approximately parallel to the C-helix backbone. **D** BR-sTnC is incorporated into demembranated muscle fibres using either an extraction and reconstitution method (Ferguson et al. 2003) or passive exchange in a relaxing solution (Knowles et al. 2012). Localisation of BR-TnC on thin filaments is verified by confocal microscopy (example image from a BR_15-22_-TnC (A-helix) reconstituted cardiac trabecula by Dr Andrea C. Knowles). **E** Polarised fluorescence from the BR-sTnC exchanged muscles in different physiological states is collected in line with the excitation optics and perpendicular to the illumination beam and the trabecular axis. **F** The intensities of the parallel and perpendicular polarisation components of each collected beam are used to calculate the order parameters < *P*_2_ > and < *P*_4_ > , which provide complementary information about the orientation of the fluorescence dipole and hence of the labelled helix with respect to the thin filament axis. In this example, the in situ orientation of the protein domain (sTnC_N_) during maximal calcium activation is calculated by combining the order parameters from multiple probes (N-, A- and C-helix) with the angular coordinates of the probes in our molecular frame extracted from a chosen crystal or NMR structure of the protein domain (NMR, PDB 1NPQ). **G** The orientation of sTnC_N_ is described in terms of two angles: *β*, the angle between the D-helix axis (dark grey) and the thin filament axis, and *γ*, which describes rotation around the D-helix. Increasing *γ* means counter clockwise rotation of the N-lobe and BR-labelled helices N (BR_6-13_, blue), A (BR_18-25_, green) and C (BR_57-64_, salmon pink) around the D-helix axis as viewed from the C-terminal end of the D-helix axis. The BR cross-linking sites are shown as spheres, whereas the dipole is the line connecting the spheres. Using a model-free maximum entropy (ME) formalism (Van der Heide et al. 2000), we calculate the smoothest distribution of the angles *β* and *γ* that is consistent with both the measured ordered parameters and the molecular fold of the chosen structure. It is represented as a contour plot with hotter colours denoting more probable orientations (replotted from Ferguson et al. 2003, highest probability *β* = 82°, *γ* = 10°)

FISS (Fig. 2) was developed to overcome that limitation and thereby to allow the determination of protein domain orientations in cells (Corrie et al. 1999). The key innovation was to synthesise a bifunctional rhodamine (BR) with cysteine-reactive groups on either end of the fluorescence dipole and attach it to a pair of cysteines on the surface of the target domain, in their case the C-terminal lobe of the myosin RLC. The cysteines were chosen to be 10–15 Å apart, for example seven residues apart on a surface α-helix. BR attachment was assumed not to perturb the conformation of the target domain, and the fluorescence dipole of BR was assumed to be parallel, on average, to the vector joining the *β*-carbons of the two cysteines, with limited fast mobility around that vector (Fig. 2C). Polarised fluorescence measurements in a cylindrically symmetric system like a muscle fibre yield information about the axial orientation of the probe dipole with respect to the long axis of the muscle cell, i.e. *β*, for each pair of cysteines. Because the probe is a dipole, the method cannot distinguish between *β* and 180°-*β*.

Corrie et al. fitted their data with a Gaussian distribution of *β*, to allow for orientational disorder of the domain on timescales slower than the fluorescence lifetime. They also used polarised fluorescence measurements on two axes orthogonal to the muscle cell axis (*x* and *y* illumination; Fig. 2E) to correct for fluorophore motion on timescales faster than the fluorescence lifetime (Dale et al. 1999). Finally, they extended the approach to a total of four cysteine pairs on the same target domain and combined the data using a crystallographic structure of the domain to constrain the angles between the vectors joining the probe attachment points. This yielded two parameters that describe the orientation of the protein domain in the muscle cell. One parameter, *β*, is the angle between a defined reference axis in the molecular frame of the protein domain and the *z* axis of the muscle fibre, and the other (*γ*) describes the rotation of the domain around the reference axis (Fig. 2F). Although this is an incomplete characterisation of the 3D orientation of the protein domain in the muscle cell because the azimuthal angle α is inaccessible in a cylindrically symmetrical system, *β* and *γ* (with their associated disorder functions) can effectively constrain molecular mechanisms of protein function.

This technique was subsequently generalised to remove the constraint of a Gaussian orientation distribution of *β* and *γ*, using maximum entropy (ME) formalism (van der Heide et al. 2000). This analytical approach determines the most likely (*β*, *γ*) orientation distribution that is consistent with the measured polarised fluorescence data from a set of bifunctional probes for a given set of relative probe orientations defined by a high-resolution structure of the domain. This approach is particularly useful when there are multiple populations of protein domains in the cell with distinct orientations. The ME orientation distribution has limited angular resolution and cannot exclude the existence of more complex distributions, particularly those containing minor populations of target domains with different orientations. The approach fails if there is *no* possible domain conformation compatible with the measured polarised fluorescence data, a result which shows that the in situ structure of the domain must be different from the in vitro structure that was used to constrain the inter-probe angles.

The FISS workflow is summarised in Fig. 2 for the example of bifunctional rhodamine cross-linking cysteines introduced at positions 57 and 64 in the C-helix of skeletal muscle sTnC_N_ (Ferguson et al. 2003). The key steps are (A) preparation of the modified sTnC with cysteines in the desired positions, (B) chemical ligation of BR with the two cysteines, (C) purification of the bifunctionally labelled product, (D) introduction of the BR-sTnC into a demembranated muscle cell to replace the native protein, (E) measurement of polarised fluorescence intensities in the muscle cell, (F) combination of the experimental data with available *in vitr*o structural information and (G) determination of the (*β*, *γ*) orientation distribution of sTnC_N_ in the muscle cell by maximum entropy analysis.

The angular resolution of FISS is limited by the dependence of the measured polarised intensities on the cosine-squared angle between the fluorescence dipole and the muscle fibre axis. Analogous to the description of a spatial distribution in terms of a series of spatial frequencies with Fourier coefficients, the probe orientation distribution can be represented in terms of a series of orientation functions called Legendre polynomials, with coefficients called order parameters. For dipole probes in a cylindrically symmetric system, only the first two even-order parameters, called < *P*_2_ > and < *P*_4_ > , can be determined by conventional polarised fluorescence measurements, although higher-order terms could in principle be determined using two-photon excitation or pulse-probe techniques (Bell et al. 2002). < *P*_2_ > and < *P*_4_ > are related to the first two even Legendre polynomials, which contain terms in *cos*^2^ and *cos*^4^, respectively, effectively limiting the angular resolution to around 20° to 30° for a single probe (van der Heide et al. 2000). This limit can be overcome by combining data from multiple bifunctional probes if the angles between the vectors linking pairs of probe attachment points are assumed to be the same as in a high-resolution structure of the target protein. In practice, there may be no advantage in using more than 4 or 5 probes, which typically gives an angular resolution approaching 10°. At that level, other limitations of the method, including the possibility that the probe dipole is not exactly parallel to the attachment sites or that the 3D structure of the target domain in the cell is not identical to that in the isolated protein in solution or in a crystal, are expected to dominate.

## Determination of the orientation of the N-terminal lobe of TnC in skeletal muscle (sTnC_N_)

**Fig. 3:**
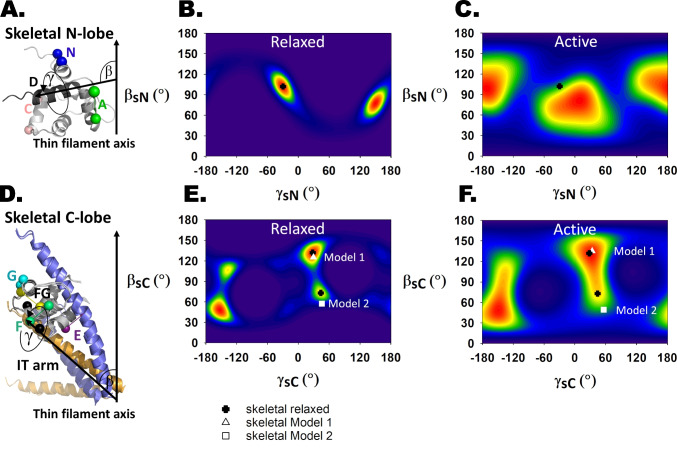
Maximum entropy analysis of skeletal muscle FISS data during relaxation and maximum calcium activation. The ME maps were replotted using data from Ferguson et al. (2003) and Knowles et al. (2012). Helices are named from the N-terminus: N, A, B, C, D, E, F and G. **A** Cartoon representation of the chicken fast skeletal N-lobe in the Ca^2+^-free or APO state (PDB 1TNP, Gagné et al. 1995). The BR dipoles on helices N (BR_6-13_), A (BR_18-25_) and C (BR_57-64_) are shown in blue, green and salmon pink, respectively. **B** ME orientation distribution of the skeletal N-lobe domain in relaxed muscle fibres based on the N-, A- and C-helix probe data and the APO NMR structure 24 from 1TNP. The two high-probability orientations are equivalent and represent the two ends of the dipole or the two sarcomere halves. Therefore, only one of them is marked with a black cross (*β* = 102°, *γ* =  − 30°). This orientation with respect to the thin filament axis (pointed end up) is shown in **A**. **C** The orientation distribution of the skeletal N-lobe during active contraction is based on the Ca^2+^-loaded NMR structure (PDB 1NPQ, sTnC_N_.2Ca^2+^.sTnI_116-132_.BR_57-64_, Mercier et al. 2003). The black cross indicates the distribution peak during relaxation for comparison (active peak *β* = 82°, *γ* = 10°). **D** Orientation of the skeletal C-lobe or IT arm during relaxation derived from troponin Model 2 by Knowles et al. (2012) (aligned APO PDB 1YV0 and Ca^2+^ PDB 1YTZ troponin IT arm domains, Vinogradova et al. 2005). The C-lobe is shown as a cartoon representation sandwiched between TnI and TnT with the following probes: E-helix (BR_97-104_) in deep purple, F-helix (BR_117-124_) in lime green, FG interhelix (BR_120-136_) in yellow and G-helix (BR_133-140_) in cyan. **E** ME orientation distribution of the skeletal C-lobe or IT arm domain during relaxation based on data from four probes (E, F, FG and G) and coordinates from PDB 1YTZ. The black crosses indicate the two peaks of the ME map (peak 1: *β* = 130°, *γ* = 30° and peak 2: *β* = 73°, *γ* = 44°). The white triangle shows the refined Gaussian solution named Model 1 (*β* = 125°, *γ* = 30°). The white square shows the other solution (Model 2, *β* = 57°, *γ* = 46°). **F** ME orientation distribution of the skeletal C-lobe in active contraction. The black crosses indicate the peaks of the distribution in relaxed muscle, whereas the white triangle and square correspond to the active conformations in Model 1 (*β* = 135°, *γ* = 34°) and Model 2 (*β* = 49°, *γ* = 56°), respectively

**Fig. 4:**
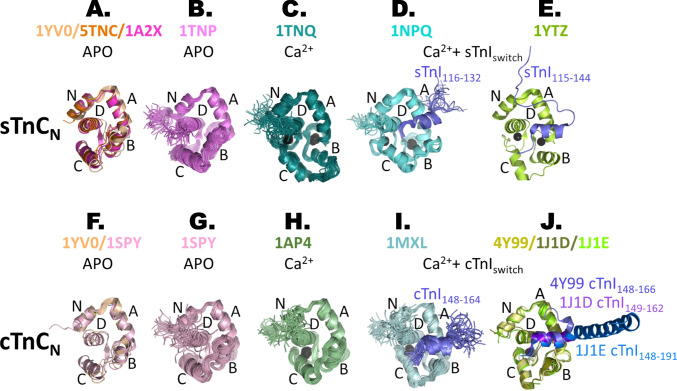
Comparison of cardiac and skeletal TnC_N_ structures in different states. The regulatory domain of troponin is viewed along helix D. **A**–**E** The Ca^2+^- and switch peptide–induced structural transitions in the skeletal N-lobe. **A** Superposition of APO crystal structures of sTnC_N_ along D-helix (EFLVMMVRQ): chicken skeletal TnC_N_ in wheat (PDB 1YV0, Vinogradova et al. 2005), turkey skeletal TnC_N_ in orange (PDB 5TNC, Herzberg and James 1985), rabbit skeletal TnC_N_ in light magenta (PDB 1A2X, Vassylyev et al. 1998). The N-lobe is in a closed conformation with a buried hydrophobic pocket. **B** Solution NMR family of structures of the APO chicken skeletal TnC_N_ in violet (PDB 1TNP, Gagne et al. 1995). The interhelical angles are similar to the crystal structures, except for the C/D angle, which is more open. **C** NMR assembly of structures of the Ca^2+^-loaded chicken skeletal TnC_N_ in deep teal (PDB 1TNQ, Gagne et al. 1995). In skeletal muscle, calcium is sufficient to induce N-lobe opening with the movement of helices B and C away from N, A and D. This exposes a hydrophobic patch to which TnI_switch_ can bind. **D** NMR assembly of structures of the Ca^2+^-and switch peptide–bound chicken skeletal TnC_N_ shown in aquamarine with BR cross-linked with C-helix (sTnC_N_.2Ca^2+^.sTnI_116-132_.BR_57-64_, PDB 1NPQ, Mercier et al. 2003). This structure is more closed than 1TNQ and the corresponding crystal structure in **E**. **E** Crystal structure of the chicken skeletal TnC_N_ (lemon green) in the presence of Ca^2+^ and TnI_115-144_ shown in slate (PDB 1YTZ, Vinogradova et al. 2005). **F**–**J** The corresponding Ca^2+^- and switch peptide–dependent rearrangements in the cardiac regulatory domain. **F** Structural alignment of APO chicken skeletal TnC_N_ in wheat (crystal structure PDB 1YV0, Vinogradova et al. 2005) and APO human cardiac TnC_N_ in light pink (NMR average structure, PDB 1SPY, Spyracopoulos et al. 1997). The structural fold is very similar with a root mean square deviation (RMSD) between the equivalent Cα atoms of 2.435 Å. **G** The NMR assembly of structures for PDB 1SPY. **H** NMR assembly of structures of Ca^2+^-bound human cardiac TnC_N_ in pale green (PDB 1AP4, Spyracopoulos et al. 1997). Binding of calcium does not open the cardiac N-lobe as in the skeletal N-lobe. **I** NMR assembly of structures of the Ca^2+^- and switch peptide–bound human cardiac TnC_N_ in pale cyan (cNTnC.Ca^2+^.cTnI_148-164_, PDB 1MXL, Li et al. 1999). Binding of the switch region of cTnI opens the cardiac domain, but it is still more closed compared to skeletal muscle. **J** Aligned crystal structures of the Ca.^2+^- and switch peptide–bound human cardiac TnC_N_ (Takeda et al. 2003): PDB 4Y99 (cTnC_N_ in pale yellow and cTnI_148-166_ in slate), PDB 1J1D (cTnC_N_ in olive green and cTnI_149-162_ in purple blue), PDB 1J1E (cTnC_N_ in chartreuse green and cTnI_148-191_ in marine blue). Although there are relatively small differences in the overall fold between the crystal structures and the average NMR structure 1MXL (RMSD ~ 1.3–1.7 Å), the interhelical B/D angle is decreased by ~ 15° in all the crystal structures, whereas the C/D angle is increased by ~ 15° in 4Y99

The orientation of sTnC_N_ in demembranated fibres from mammalian skeletal muscle was determined by FISS using bifunctional rhodamine probes on three surface-accessible helices (Fig. 3A (Ferguson et al. 2003)). In relaxing conditions, when [Ca^2+^] is sufficiently low that no Ca^2+^ is bound to the regulatory sites of sTnC_N_, the ME orientation map (Fig. 3B) was calculated from the measured order parameters using an NMR structure of Ca^2+^-free or APO sTnC_N_ (1TNP (Gagne et al. 1995); Figs. 3A and 4B). The orientation distributions in Fig. 3 are represented as contour maps of the probability of the domain being in that (*β*, *γ*) orientation, with red indicating the highest and dark blue the lowest probability. The two red regions are equivalent and can be regarded as orientations along the dipole pointing in opposite directions; the dipole orientation (*β*, *γ*) is equivalent to (180°-*β*, *γ* ± 180°). The orientation distribution of sTnC_N_ in relaxed muscle is very narrow and centred on *β*_N_ = 102°, *γ*_N_ =  − 30° and its dipole-related equivalent.

In contrast with the narrow orientation distribution for sTnC_N_ calculated using the 1TNP NMR structure, maximum entropy analysis of the same set of FISS data produced no solution when the relative angles between the pairs of cysteines were constrained using two crystal structures of sTnC_N_ in the APO state (5TNC (Herzberg and James 1985); 1A2X (Vassylyev et al. 1998); Fig. 4A). These two crystal structures are more closed than the NMR structure, as is the APO structure of sTnC_N_ determined subsequently in crystals of the troponin core domain (1YV0 (Vinogradova et al. 2005); Fig. 4A). The FISS results show that these more closed structures are not formed in the native environment of a skeletal muscle cell. In the case of sTnC_N_ in a relaxed muscle cell, therefore, FISS not only determined a well-defined in situ (*β*_N_, *γ*_N_) orientation but also showed that the in situ fold of sTnC_N_ is inconsistent with the more closed structures determined by crystallography.

The orientation of sTnC_N_ was also determined by FISS in actively contracting muscle, by combining the order parameters measured during contraction with an NMR structure of sTnC_N_ with both calcium and the TnI switch peptide bound (1NPQ (Mercier et al. 2003); Fig. 4D). Moreover, this NMR structure was determined using sTnC_N_ in which bifunctional rhodamine cross-linked one of the pairs of surface-accessible cysteines used for FISS, showing that attachment of bifunctional rhodamine at these sites does not change the fold of the sTnC_N_ backbone. In actively contracting muscle fibres, the peak value of *β*_N_ decreased by 20° and *γ*_N_ increased by 40° (Fig. 3C) with respect to their values in relaxed muscle (black cross), and the orientation distribution became broader.

The change in the orientation of the sTnC_N_ C-helix probe on activation of skeletal muscle is particularly large and was used subsequently for more detailed studies of the effects of calcium and myosin binding on the orientation of sTnC_N_ (Sun et al. 2006; Brunello et al. 2023). The more recent study (Brunello et al. 2023) was carried out at 26 °C with the filament lattice osmotically compressed to its physiological spacing, and using a milder TnC exchange protocol, conditions in which myosin filament-based regulation, is preserved (Fusi et al. 2015; Caremani et al. 2019). The sTnC_N_ C-helix orientation during full calcium activation was unaffected by the complete abolition of active force and strong binding of myosin motors to actin by 25 µM blebbistatin, showing that the calcium-bound conformation of sTnC_N_ is insensitive to myosin binding to the thin filament (Brunello et al. 2023). Even the induction of rigor, a non-physiological state in which ATP is absent and myosin motors bind tightly to the thin filament, did not produce a further change in sTnC_N_ C-helix orientation beyond that seen in active contraction at the same calcium concentration (Sun et al. 2006).

The calcium sensitivity of the orientation change of the sTnC_N_ C-helix probe was determined using steady-state titrations with EGTA/Ca^2+^-EGTA buffers. The results of such titrations are typically fitted with the Hill equation *Y* = 1/(10^*n*H(pCa−pCa50)^), where pCa_50_ is the pCa corresponding to the half-maximum change in *Y*, and *n*_H_ is the Hill coefficient, which gives a measure of the cooperativity of the process. pCa_50_ for the orientation change of the sTnC_N_ C-helix on activation of skeletal muscle in near-physiological conditions decreased by about 0.3 units when active force was abolished by blebbistatin (Brunello et al. 2023), so the calcium-dependent orientation change in sTnC_N_ is sensitised by myosin binding although the overall amplitude of the change is not affected. *n*_H_ was about 2.5 and was also unaffected by the inhibition of active force. Activation of the thin filament is intrinsically cooperative, but this cooperativity is not due to myosin binding (Sun et al. 2006). Force itself has a higher cooperativity (*n*_H_ = 3.6) in these conditions, because of the additional cooperativity contributed by the activation of the myosin filaments (Brunello et al. 2023; Brunello and Fusi 2023).

## Determination of the orientation of the C-terminal lobe of TnC in skeletal muscle

An analogous approach was applied to the C-terminal lobe of TnC in skeletal muscle (sTnC_C_) (Knowles et al. 2012) using the crystal structure of the troponin core complex in the Ca^2+^-bound state (1YTZ (Vinogradova et al. 2005); Fig. 3D) to constrain the inter-probe angles. The latter was chosen for this ME analysis in preference to the APO structure (PDB 1YV0 (Vinogradova et al. 2005)) because residues 87–97 of the sTnC_C_ E-helix were not resolved in the APO structure, although the overall IT arm fold is almost identical in the two structures (Fig. 3D). Because sTnC_C_ is tightly bound to TnI and TnT in the IT arm of the troponin core complex, the reference axis for this analysis was defined as the long axis of the coiled-coil between TnI and TnT (Fig. 3D).

The ME distribution of (*β*_C_, *γ*_C_) calculated from the FISS data from the sTnC_C_ probes in relaxed skeletal muscle (Fig. 3E) was unexpectedly complicated, with two peaks centred on *β*_C_ = 130°, *γ*_C_ = 30° and *β*_C_ = 75°, *γ*_C_ = 45°, each with its dipole-related equivalent. It was not possible to distinguish between these two solutions by polarised fluorescence with the set of four bifunctional probe sites used in that study. Moreover, by exploring specific Gaussian orientation models with refinement of inter-probe angles, Knowles et al. showed that there were two solutions, each centred on an orientation close to one of the peaks in the ME map and with a Gaussian angular dispersion of about 20° that gave equally good fits to the FISS data in all the conditions studied. Later studies, using FISS data from the highly homologous IT arm of cardiac muscle troponin in heart muscle cells (Sevrieva et al. 2014) and cryo-EM structures of the cardiac thin filament (Yamada et al. 2020; Risi et al. 2021b, 2024) discussed below, showed that the solution that Knowles et al. called ‘Model 2’ was likely to be correct.

The peak values of (*β*_C_, *γ*_C_) obtained from the refined Gaussian Model 2 of Knowles et al. were slightly different between relaxation and active contraction, showing that the IT arm does tilt (*β*_C_ decreases from 57° to 49°) and twist (*γ*_C_ increases from 46° to 56°) slightly on activation. This small but reproducible re-orientation of the IT arm is primarily due to the binding of myosin to the thin filament, because the angle changes were much reduced when active force was inhibited by a small molecule, *N*-benzyl-p-toluene sulphonamide (BTS), that prevents myosin binding to actin (Knowles et al. 2012).

**Fig. 5:**
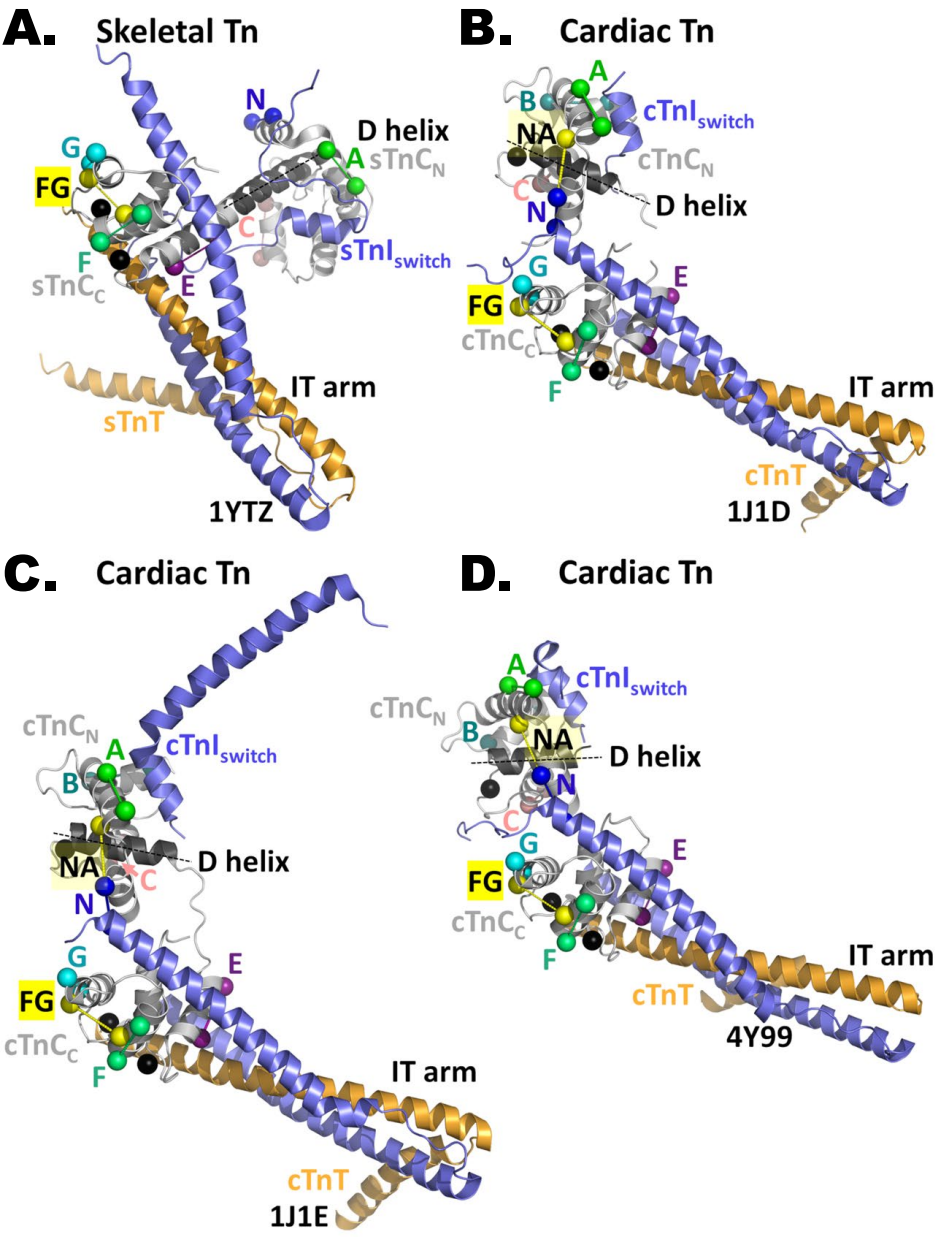
Troponin core domain structures in the Ca^2+^-bound state. TnC is depicted in grey, TnI in slate and TnT in bright orange. The IT arm in the different Tn complexes is oriented as in the active state according to Model 2 for skeletal Tn (Knowles et al. 2012) and Sevrieva et al. (2014) for cardiac muscle, whereby the thin filament axis is vertical with the pointed end upward as in Fig. 3. The D-helix is depicted in dark grey, and the line indicates that it is at a different angle with respect to the thin filament in the different complexes. **A** Skeletal troponin structure sTnC_5-163_.4Ca^2+^.sTnI_4-144_.sTnT_160-248_ (PDB 1YTZ, Vinogradova et al. 2005). The probe dipoles are shown for the N-lobe: N-helix (BR_6-13_) in blue, A-helix (BR_18-25_) in green and helix C (BR_57-64_) in blue. C-lobe probe dipoles: E-helix (BR_97-104_) in deep purple, F-helix (BR_117-124_) in lime green, FG interhelix (BR_120-136_) in yellow and G-helix (BR_133-140_) in cyan. **B** Cardiac troponin structure cTnC_2-90, 93–161_.3Ca^2+^.cTnI_37-138, 149–162_.cTnT_200-274_ (PDB 1J1D, Takeda et al. 2003). N-lobe probes: N-helix (BR_3-10_) in salmon pink, NA interhelix (BR_10-17_) in yellow, A-helix (BR_15-22_) in green, B-helix (BR_39-46_) in deep teal and helix C (BR_55-62_) in blue. C-lobe probe dipoles: E-helix (BR_95-102_) in deep purple, F-helix (BR_115-122_) in lime green, FG interhelix (BR_118-134_) in yellow and G-helix (BR_131-138_) in cyan. **C** Cardiac troponin structure cTnC_1-48, 51–161_.3Ca^2+^.cTnI_40-136, 148–191_.cTnT_202-276_ (PDB 1J1E, Takeda et al. 2003). **D** Cardiac troponin structure cTnC_1-83, 89–161_.3Ca.^2+^.cTnI_31-136, 148–166_.cTnT_199-272_ (PDB 4Y99, Takeda et al. 2003)

The relative orientations of sTnC_N_ and sTnC_C_ determined by FISS (Ferguson et al. 2003; Knowles et al. 2012) are not consistent with the central D- and E-helices of sTnC being colinear, as observed in crystal structures of both isolated sTnC (5TNC (Herzberg and James 1985)) and the core complex of skeletal muscle troponin (1YV0, 1YTZ (Vinogradova et al. 2005); Fig. 5A). The comparison provides further evidence that the tertiary structure of troponin seen in a crystal can be dramatically different from that in the native environment of the cell. The D/E-helix is also broken in cardiac troponin, in that case as also shown by crystallography (1J1D, 1J1E, 4Y99 (Takeda et al. 2003); Fig. 5B–D), by cryo-EM (Oda et al. 2020; Yamada et al. 2020; Risi et al. 2021b, 2024) and by FISS as described below.

A single bifunctional probe bound to the E-helix of sTnC_C_ provides a sensitive measure of changes in the orientation of the IT arm (Sun et al. 2006; Knowles et al. 2012; Brunello et al. 2023). Calcium titrations in near-physiological conditions in which thick filament regulation is preserved showed that the change in orientation of the sTnC_C_ E-helix has two components, a smaller component linked to Ca^2+^ binding and a larger component linked to myosin binding. The latter component has the same calcium sensitivity as active force and a much higher cooperativity than the calcium component (Brunello et al. 2023).

The three regulatory states of the thin filament signalled by the orientation of the E-helix of TnC on activation of skeletal muscle in near-physiological conditions have some analogy to the blocked, closed and open states defined by ATPase assays using isolated thin filaments and myosin head domains (McKillop and Geeves 1993) and by electron microscopy of those components (Lehman et al. 1994). The blocked state corresponds to the structure of the thin filament in relaxed muscle, and the closed state to that with Ca^2+^ bound to the regulatory sites of TnC when myosin binding is prevented. The open state in previous biochemical and EM studies was represented by the rigor (ATP-free) state, which might be distinct from the structure during active contraction in the presence of ATP. However, Brunello et al. (2023) showed that the conformation of troponin during active contraction in near-physiological conditions signalled by a FISS probe on the E-helix of sTnC_C_ is distinct from that in either relaxation (blocked) or with Ca^2+^ bound to the regulatory sites of TnC when myosin is inhibited (closed). Moreover, the order parameters for the E-helix probe during active contraction in near-physiological conditions in the region of the thin filament that overlaps with the myosin filaments (Brunello et al. 2023) are not significantly different from those measured in rigor in the presence of Ca^2+^ (Ferguson et al. 2003; Sun et al. 2006). Thus, to a first approximation, the conformation of the IT arm of troponin during active contraction in the myosin overlap zone of skeletal muscle sarcomeres seems to be similar to that of the open state described in vitro. This conclusion is consistent with that reached for the azimuthal position of tropomyosin during active contraction of demembranated fibres from skeletal muscle on the basis of X-ray diffraction measurements (Bershitsky et al. 2017).

The FISS results from the sTnC_C_ E-helix probe in skeletal muscle therefore support the hypothesis that calcium and myosin have additive contributions to thin filament activation. The existence of the myosin component leads to a positive feedback loop, in which myosin binding promotes further myosin binding, and so on. In the intact sarcomere, this works in parallel with a second positive feedback loop in which myosin filaments and motors are activated by stress (Linari et al. 2015). Together, these two feedback loops act to make the activation of skeletal muscle highly cooperative (Brunello et al. 2023; Brunello and Fusi 2023).

## Time-resolved FISS studies on TnC in skeletal muscle

The results described above, like most previous studies of the calcium dependence of muscle contraction, were obtained in the steady state, i.e. at constant [Ca^2+^]. That condition is far removed from the transient activation that occurs in intact muscle cells; in mammalian fast-twitch skeletal muscle in physiological conditions, the half width of the intracellular free calcium transient is only a few milliseconds (Baylor and Hollingworth 2003). It is therefore important to extend FISS studies to the physiological timescale. For muscle activation, this can be achieved using photolytic release of calcium from caged calcium, combined with time-resolved FISS (Fusi et al. 2014; Brunello et al. 2023). The more recent study, carried out in conditions that preserve myosin filament-based regulation (Brunello et al. 2023), showed that the response of both lobes of sTnC to a step increase in [Ca^2+^] has a very fast component, with a rate constant of about 5000 s^−1^ at 26 °C. Both the regulatory head and the IT arm of troponin therefore respond to Ca^2+^ binding to the regulatory sites with negligible delay on the physiological timescale of the calcium transient.

The response of the IT arm signalled by the sTnC_C_ E-helix probe also had a slower component, with a rate constant close to that of force development, consistent with the idea that binding of myosin motors to the thin filament switches it further on. Moreover, this component was abolished in the presence of blebbistatin, which stabilises the off state of the thick filament and prevents motor binding to actin and active force generation. The responses of the IT arm to calcium and myosin binding are therefore clearly distinguishable kinetically, on the physiological timescale, as well as in steady-state calcium titrations.

## Determination of the orientation of TnC in heart muscle by FISS

**Fig. 6:**
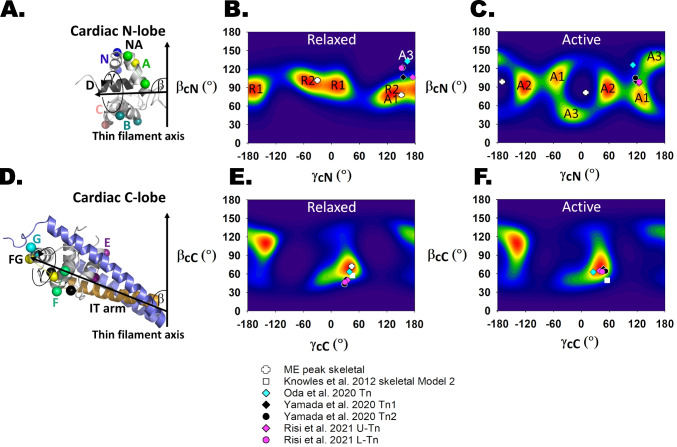
Maximum entropy analysis of cardiac muscle FISS data during relaxation and maximum calcium activation. The ME maps were replotted using data from Sevrieva et al. (2014). **A** Cartoon representation of the human cardiac N-lobe in its Ca^2+^-free or APO R1 orientation (PDB 1SPY, Spyracopoulos et al. 1997; Sevrieva et al. 2014). The BR dipoles on helices N (BR_3-10_), NA (BR_10-17_), A (BR_15-22_), B (BR_39-46_) and C (BR_55-62_) are shown in blue, yellow, green, deep teal and salmon pink, respectively. **B** ME orientation distribution of the cardiac N-lobe domain in relaxed trabeculae based on the N-, NA-, A-, B- and C-helix probe data and the APO NMR average structure (PDB 1SPY). There are two high-probability orientations labelled R1 and R2 (see Table 1). Their equivalent peaks are also shown. The white cross indicates the physiological state-matched orientation in skeletal muscle. Calculated state-matched *β* and *γ* from available cryo-EM fitted structures (Table 1): black diamond (Tn1, Yamada et al. 2020), black circle (Tn2, Yamada et al. 2020), cyan diamond (Oda et al. 2020), magenta diamond (U-Tn, Risi et al. 2021b), magenta circle (L-Tn, Risi et al. 2021b). A1 and A3 represent active orientations from the ME map in **C**. **C** ME orientation distribution of the cardiac N-lobe domain during active contraction based on the N, NA, A, B and C-helix probe data and the NMR average structure of the human cardiac Ca.^2+^-bound TnC_N_.cTnI_148-164_ complex (PDB 1MXL, Li et al. 1999). There are three active orientation pairs A1, A2 and A3 (Table 1). **D** Cartoon representation of the cardiac IT arm in relaxed trabeculae showing C-lobe probe locations: E-helix (BR_95-102_) in deep purple, F-helix (BR_115-122_) in lime green, FG interhelix (BR_118-134_) in yellow and G-helix (BR_131-138_) in cyan (PDB 1J1D, Takeda et al. 2003). **E** ME map of the in situ C-lobe orientations in relaxed cardiac muscle based on the E-, F-, FG- and G-helix probe data and PDB 1J1D/1J1E (*β* and *γ* in Table 1). The white square shows the state-matched Model 2 orientation in skeletal muscle (Knowles et al. 2012). **F** ME map of the cardiac TnC_C_ orientations during active contraction based on the in situ E-, F-, FG- and G-helix probe data and PDB 1J1D/1J1E (*β* and *γ* in Table 1)

The orientation of the N-lobe of the cardiac isoform of TnC (cTnC_N_) was determined by FISS in trabeculae isolated from the right ventricle of rat heart using five different bifunctional probes (Fig. 6A (Sevrieva et al. 2014)), and an NMR structure of cTnC_N_ without Ca^2+^ bound to the regulatory sites (1SPY (Spyracopoulos et al. 1997); Fig. 4G). The maximum entropy map for relaxing conditions (Fig. 6B) showed that the preferred cTnC_N_ orientation (red region) has *β*_N_ around 100°, similar to its value in skeletal muscle, but with a much wider range of *γ*_N_. The distribution can be described in terms of two sub-peaks with the same *β*_N_ centred on *γ*_N_ = 10° (R1) and − 50° (R2), bracketing the value in skeletal muscle (*white* cross).

The orientation distribution of cTnC_N_ during maximal calcium activation (Fig. 6C) was determined by FISS using the same five probes, combined with an NMR structure of cTnC_N_ with both calcium and the switch peptide bound (1MXL (Li et al. 1999); Fig. 4I). This distribution has three sub-peaks, one at *β*_N_ = 110°, *γ*_N_ =  − 50° (A1), close to the R2 relaxed sub-peak, and two new sub-peaks at *β*_N_ = 85°, *γ*_N_ = 55° (A2) and *β*_N_ = 40°, *γ*_N_ =  − 20° (A3). None of these three sub-peaks is at the same orientation as that in skeletal muscle at maximal calcium activation (*white* cross). The orientation distribution determined during maximal calcium activation of heart muscle in the presence of blebbistatin was almost identical to this, indicating that the difference between the relaxed and active distributions is almost entirely due to calcium rather than myosin binding. No orientation of the crystal structure of the cardiac isoform of cTnC_N_ with bound calcium (1J1D, 1J1E, 4Y99 (Takeda et al. 2003); Fig. 5B, C) was consistent with the FISS data. We conclude that cTnC_N_ is more open in situ than in the crystal, as in the case of skeletal muscle sTnC_N_ in the relaxed state (Ferguson et al. 2003). The same conclusion was recently drawn from a cryo-EM structure of the cardiac thin filament without bound Ca^2+^ (Risi et al. 2024).

In contrast with the multi-peak orientation distributions determined by FISS for the N-lobe of cTnC, a simpler distribution was obtained for the C-lobe (cTnC_C_) using a set of four FISS probes and the crystal structure of the IT arm in the Ca^2+^-bound state (1J1D, 1J1E, 4Y99 (Takeda et al. 2003); Fig. 4J). In relaxing conditions (Fig. 6E), there was a single peak in the distribution at *β*_C_ = 69°, *γ*_C_ = 39°. The orientation during maximal calcium activation of heart muscle was almost identical: *β*_C_ = 66°, *γ*_C_ = 40° (Fig. 6F). These orientations are close to the Model 2 solutions for skeletal muscle: *β*_C_ = 57°, *γ*_C_ = 46° in relaxation (Fig. 6E *black* cross (Knowles et al. 2012)) and *β*_C_ = 49°, *γ*_C_ = 56° during maximal calcium activation (Fig. 6F, *white* square). The structure of the IT arm is almost identical in crystal structures of the core domains of skeletal muscle troponin (Fig. 5A (Vinogradova et al. 2005)) and heart muscle troponin (Fig. 5B–D (Takeda et al. 2003)).

Following the approach used in skeletal muscle, FISS probes on the cTnC_N_ C-helix and cTnC_C_ E-helix were used for more detailed studies of the effects of calcium and myosin binding to thin filaments on the orientation of cTnC_N_ and the IT arm in heart muscle cells (Sun et al. 2009; Zhang et al. 2017), although not in the near-physiological conditions used for the analogous studies on skeletal muscle. The change in the mean orientation of the cTnC_N_ C-helix on activation of heart muscle is much smaller than that in skeletal muscle, as indicated by the change in the order parameter < *P*_2_ > on maximal calcium activation, which is about a quarter of that in skeletal muscle (Sun et al. 2009). This smaller mean orientation change partly reflects the more ordered conformation of TnC_N_ in relaxed skeletal muscle (Fig. 3B), compared with the broader distribution in heart muscle (Fig. 6B). The steady-state calcium dependence of < *P*_2_ > for the cTnC_N_ C-helix probe was, however, similar to that in skeletal muscle, approximately tracking active force with a Hill coefficient (*n*_H_) of about 3. The effect of pharmacological abolition of active force on the orientation of the cTnC_N_ C-helix was also similar to that described above for skeletal muscle; there was no effect on the amplitude of the orientation change induced by calcium binding, but the calcium sensitivity was higher in the presence of force-generating myosin motors.

The change in the mean orientation of the C-lobe of cTnC, reported by that of the cTnC_C_ E-helix on activation of heart muscle, was also smaller than that observed in skeletal muscle. It had a similar dependence on calcium concentration, also with a Hill coefficient (*n*_H_) of about 3, and the abolition of active force reduced both the amplitude of the orientation change and its calcium sensitivity. Thus, as in skeletal muscle, the IT arm of heart muscle troponin tilts slightly in response to the binding of force-generating myosin motors in addition to the effect of calcium binding to TnC_N_. A larger additional tilt of the E-helix was observed when heart muscle cells were put into rigor by ATP depletion, and this rigor orientation was indistinguishable from that in skeletal muscle. In skeletal muscle, the orientation of the IT arm of troponin reported by that of the E-helix was similar during maximal activation and in rigour. Since rigor is considered to be a model for the fully activated or open state of the thin filament, that comparison suggested that thin filaments are in the open state during maximal activation of skeletal muscle. The same conclusion does *not* hold for cardiac muscle, in which the orientation of the E-helix during maximal calcium activation corresponds to a much less activated state of the thin filament. This conclusion would apply even more strongly to the activation of the heart in physiological conditions, in which thin filaments are only about half-maximally activated by calcium.

Physiologically, the contractility of heart muscle is modulated by additional factors that alter the calcium sensitivity of troponin in situ. Increasing the length of heart muscle cells or sarcomeres increases calcium sensitivity and maximal force, in a phenomenon called length-dependent activation that is thought to be the cellular basis of the Frank-Starling relationship that links the ejection volume of the heart to venous filling (de Tombe et al. 2010). The cTnC_N_ C-helix and cTnC_C_ E-helix probes provided tools to characterise the in situ changes in troponin conformation associated with length-dependent activation (Zhang et al. 2017). They have also been used to describe the effects of binding of the N-terminal domains of myosin binding protein-C (MyBP-C) to the thin filament (Kampourakis et al. 2014, 2018), and the effects of phosphorylation of several thin and thick filament proteins downstream of neurohumoral signalling pathways in the heart, including MyBP-C (Ponnam et al. 2019), the myosin regulatory light chain (RLC (Kampourakis et al. 2016)) and TnI (Sevrieva et al. 2023).

## Comparison with troponin domain orientations determined by cryo-EM

Early attempts to determine the conformation of the troponin core domain on the thin filaments by cryo-EM with single particle reconstruction (Pirani et al. 2006; Paul et al. 2009; Paul et al. 2017; Yang et al. 2014) led to conflicting interpretations, even at the level of the polarity of the troponin monomers on the thin filament. Subsequent technical improvements in cryo-EM provided density maps of isolated reconstituted cardiac thin filaments at higher resolution (Oda et al. 2020; Yamada et al. 2020), at both very low and saturating [Ca^2+^]. Similar and higher resolution density maps were subsequently obtained from native cardiac thin filaments at more physiological [Ca^2+^] values (Risi et al. 2021b, 2024). In the most recent structure, both the long helices of TnT and TnI in the IT arm and the shorter helices in the N-lobe of cTnC could be clearly identified, and the density of the N-lobe was well fitted by the 1SPY NMR structure (Spyracopoulos et al. 1997).

**Fig. 7:**
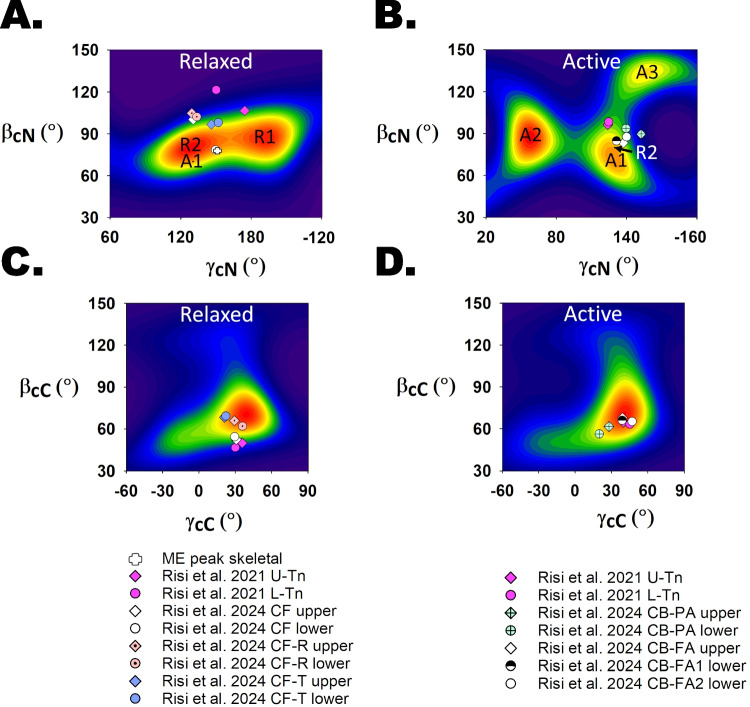
Central sections of maximum entropy maps of cardiac muscle FISS data during relaxation and maximum calcium activation showing the cryo-EM conformations from Risi et al. (2024). The ME maps were replotted using data by Sevrieva et al. (2014). **A** ME orientation distribution of the cardiac N-lobe domain in relaxed trabeculae. The white cross indicates the physiological state-matched orientation in skeletal muscle. Calculated *β* and *γ* from the cryo-EM fitted structures by Risi et al. (2021b) and Risi et al. (2024) (Table 2): magenta diamond (U-Tn, Risi et al. 2021b), magenta circle (L-Tn, Risi et al. 2021b), white diamond (Ca^2+^-free upper or CF U-Tn, Risi et al. 2024), white circle (Ca^2+^-free lower or CF L-Tn, Risi et al. 2024), light pink orange dotted diamond (Ca^2+^-free rotated upper or CF-R U-Tn, Risi et al. 2024), light pink orange dotted circle (Ca^2+^-free rotated lower or CF-R L-Tn, Risi et al. 2024), cornflower blue diamond (Ca^2+^-free tilted upper or CF-T U-Tn, Risi et al. 2024) and cornflower blue circle (Ca^2+^-free tilted lower or CF-T L-Tn, Risi et al. 2024). A1 is the active orientation from the ME map in **B**. **B** ME orientation distribution of the cardiac N-lobe domain during active contraction. The three active orientations A1, A2 and A3 are shown as well as the calculated *β* and *γ* from the cryo-EM fitted structures of Risi et al. (2021b) and Risi et al. (2024) (Table 2): magenta diamond (U-Tn, Risi et al. 2021b), magenta circle (L-Tn, Risi et al. 2021b), mint crossed diamond (Ca^2+^-bound partially active upper or CB-PA U-Tn, Risi et al. 2024), mint crossed circle (Ca^2+^-bound partially active lower or CB-PA L-Tn, Risi et al. 2024), white diamond (Ca^2+^-bound fully active upper or CB-FA U-Tn, Risi et al. 2024), semi-filled black and white circle (Ca^2+^-bound fully active 1 lower or CB-FA1 L-Tn, Risi et al. 2024) and white circle (Ca.^2+^-bound fully active 2 lower or CB-FA2 L-Tn, Risi et al. 2024). **C**, **D** ME maps of the in situ C-lobe orientations in relaxed and active cardiac muscle, respectively (*β* and *γ* in Table 2)

The orientation parameters *β*_C_ and *γ*_C_ determined by fitting the crystal structure of the IT arm into these cryo-EM density maps are close to the peaks of the orientation distributions determined in cardiac muscle cells by FISS (Figs. 6E, F and 7C, D; Tables 1 and 2). The agreement is excellent for high [Ca^2+^] and active contraction (Figs. 6F and 7D), where the differences are within the resolution of the methods and the conformational disorder that is present. Note that there are multiple symbols for the cryo-EM-based orientations corresponding to troponins on opposite strands of the thin filament and other distinct conformational populations. In the case of low [Ca^2+^] and relaxed trabeculae (Figs. 6E and 7C), the values of *β*_C_ and *γ*_C_ determined from cryo-EM data are generally smaller than those corresponding to the peak of orientation distribution determined by FISS (Tables 1 and 2), although there is considerable overlap. We conclude that the orientations distribution of the IT arm calculated by FISS and cryo-EM are the same within the uncertainties of the techniques and differences in experimental conditions.

**Table 1:** Comparison of the tilt and twist angles *β* and *γ* in degrees obtained by cryo-EM and FISS, showing cryo-EM results from Yamada et al. (2020) (6KN7 and 6KN8), Risi et al. (2021b) (7KO4 and 7KO5) and Oda et al. (2020) (6KLL, 6KLP, 6KLT, 6KLN, 6KLQ, 6KLU). The pointed end of actin is along positive *z*. The peaks of the maximum entropy orientation distributions (Sevrieva et al. 2014) are shown for both directions along the dipole (*β* and *γ*; 180°-*β* and *γ*-180°)

		Yamada et al. (2020)		Risi et al. (2021b)		Oda et al. (2020)	Sevrieva et al. (2014)	
		Tn1	Tn2	U-Tn	L-Tn	Tn Oda	*Β*, *γ*	180°-*β*, *γ* ± 180°
**N APO**	*β*	107	122	107	122	133	R1 = 85, R2 = 80	R1 = 95, R2 = 100
	*γ*	154	152	174	150	163	R1 = − 170, R2 = 130	R1 = 10, R2 = − 50
**N Ca**^**2+**^	*β*	100	105	96	98	126	A1 = 70, A2 = 85, A3 = 40	A1 = 110, A2 = 95, A3 = 140
	*γ*	118	116	124	125	111	A1 = 130 A2 = 55, A3 = − 20	A1 = − 50, A2 = − 125, A3 = 160
**C APO**	*β*	51	44	50	47	64	69	111
	*γ*	32	29	36	30	39	39	-141
**C Ca**^**2+**^	*β*	68	64	65	63	63	66	114
	*γ*	48	52	40	45	26	40	-140

**Table 2:** Comparison of the tilt and twist angles *β* and *γ* in degrees obtained by cryo-EM and FISS, showing cryo-EM results from Risi et al. (2024). PDB 8UWW for CF (Ca^2+^-free) upper (U-Tn), PDB 8UWY for CF-R (Ca^2+^-free rotated) U-Tn, PDB 8UWX and for CF-T (Ca^2+^-free tilted) U-Tn, PDB 8UYD for CF lower (L-Tn), PDB 8UZ5 for CF-R L-Tn, PDB 8UZ6 for CF-T L-Tn, PDB 8UZX for CB-FA (Ca^2+^-bound full activation) U-Tn, PDB 8UZY for CB-PA (Ca^2+^-bound partial activation) U-Tn, PDB 8V01 for CB-FA1 L-Tn, PDB 8V0I for CB-FA2 L-Tn, PDB 8V0K for CB-PA L-Tn. For comparison the *β* and *γ* angles from Risi et﻿ al. (2021b) and Sevrieva et al. (2014) are also shown

		Risi et al. (2021b**)**		Risi et al. (2024)						Sevrieva et al. (2014)	
		U-Tn	L-Tn	U-Tn			L-Tn			*β*, *γ*	180°-*β*, *γ* ± 180°
**N APO**	*β*	107	122	**CF** 100	**CF-R** 105	**CF-T** 97	**CF** 78	**CF-R** 102	**CF-T** 98	**R1** = 85, **R2** = 80	**R1** = 95, **R2** = 100
	*γ*	174	150	130	129	146	149	133	152	**R1** = − 170, **R2** = 130	**R1** = 10, **R2** = − 50
**N Ca**^**2+**^	*β*	96	98	**CB-PA** 90		**CB-FA** 83	**CB-PA** 94	**CB-FA1** 85	**CB-FA2** 88	**A1** = 70, **A2** = 85, **A3** = 40	**A1** = 110, **A2** = 95, **A3** = 140
	*γ*	124	125	163		137	140	132	140	**A1** = 130, **A2** = 55, **A3** = − 20	**A1** = − 50, **A2** = − 125, **A3** = 160
**C APO**	*β*	50	47	**CF** 53	**CF-R** 66	**CF-T** 68	**CF** 55	**CF-R** 62	**CF-T** 69	69	111
	*γ*	36	30	31	29	21	29	36	22	39	− 141
**C Ca**^**2+**^	*β*	65	63	**CB-PA** 62		**CB-FA** 68	**CB-PA** 56	**CB-FA1** 66	**CB-FA2** 65	66	114
	*γ*	40	45	28		39	20	39	47	40	− 140

In marked contrast with the agreement between cryo-EM and FISS on the orientation of the IT arm, the cTnC_N_ orientations determined by the cryo-EM studies do not reproduce the multiple peaks of the orientation determined by FISS in trabeculae, in either the low [Ca^2+^]/relaxed state (Figs. 6B and 7A), in which *β*_N_ is systematically higher in the cryo-EM orientations, or high [Ca^2+^]/active contraction state (Figs. 6C and 7B), in which the cryo-EM orientations cluster around the FISS A1 peak. Possible explanations for these differences are discussed in the following section.

## Relative advantages and limitations of FISS and cryo-EM

FISS was developed in the late 1990s with the primary aim of determining the orientations of protein domains in the native environment of muscle cells and on the physiological timescale. At that time, structures of isolated protein domains of interest were available from crystallography and NMR, but relatively little was known about how those domains were organised in their cellular environment. Electron microscopy methods of the time had insufficient resolution to answer the key mechanistic questions, and interpretation of EM images was limited by artefacts associated with fixation and staining. In general, there was a methodological gap between structural studies on isolated proteins and functional studies on cells. FISS went some way to filling that gap for the favourable case of the oriented array of filaments in muscle. The structural information available from FISS is generally limited to two angles, but because changes in those angles can be followed in a working muscle cell in response to a wide range of experimental interventions, FISS results can distinguish between alternative mechanistic hypotheses, as first shown for the regulatory light chain region of myosin (Corrie et al. 1999) and subsequently for troponin as summarised in this review.

The great strength of FISS is that it provides a method to follow structural transitions and their functional effects in intact cells on the physiological timescale. Because it is a ratiometric method, the orientation parameters determined by FISS are relatively insensitive to sample movement and extent of protein labelling. If two populations of proteins with different orientations are present, the measured orientation parameters are a linear combination of those from each population. Because the method intrinsically averages the parameters from the very large numbers of labelled domains in the cell, the method is extremely sensitive, allowing the use of relatively low illumination intensity and small samples with high time resolution. The measured order parameters are highly reproducible between different biological replicates.

However, FISS has some significant limitations. The available structural information is limited to two angles, as noted above, compared with the thousands of atomic coordinates typically determined by crystallography, NMR and increasingly by cryo-EM. Interpretation of FISS data from multiple cysteine pairs in terms of domain orientations also requires a high-resolution structural model of the domain to constrain the angles between the vectors joining the cysteine pairs. This is both a limitation, in that the FISS results depend on which structural model is used, and a strength, because they show which if any of the available structural models is compatible with the in situ structure of the domain in muscle. For example, this type of analysis showed that the fold of TnC_N_ at low [Ca^2+^] in both skeletal and cardiac muscle is more open than in the published crystal structures of the isolated domain but is consistent with both NMR solution structures and recent cryo-EM data.

FISS also requires replacement of the target protein in a cell with a recombinant protein in which a pair of surface-accessible residues has been replaced by cysteines which have been cross-linked with a bifunctional rhodamine (Fig. 2). Such replacement is only feasible after permeabilisation of the surface membrane of the muscle cell, and only for a limited set of target proteins, currently including troponin C, myosin light chains and whole troponin. Those proteins can be exchanged in relatively mild conditions that preserve the native function of the muscle cells, as assessed by the control measurements described below. Actin, myosin heavy chain, titin and MyBP-C have not so far been targeted for FISS, although novel methods of replacing protein domains in cells (Napierski et al. 2020) may make this possible in future.

Either the mutagenesis, the probe labelling or the protocol used to replace the native by the labelled protein might alter its structure or function, potentially invalidating the FISS results. Although FISS has generally been applied to target proteins of known structure, allowing rational selection of the cysteines to be replaced, it remains possible that mutagenesis changes the structure of the target protein or its interaction with another protein in the muscle cell. Such effects have been assessed by structural and functional control experiments. The most extensive structural controls have been made for the isolated E57C/E64C mutant of TnC_N_ from skeletal muscle cross-linked with bifunctional rhodamine (Fig. 2B, C). The NMR structure of this domain, including the integrity of the labelled helix and the binding of the switch peptide of TnI, was unaffected by the mutagenesis or labelling with two different bifunctional rhodamines (Mercier et al. 2003; Julien et al. 2007). Molecular dynamics calculations (De Simone et al. 2008) gave further insight into this result, showing how the flexible linkers between the fluorophore and the cysteine attachments allow conformational flexibility of the bifunctional rhodamine probe whilst confining its mean orientation to be roughly parallel to the vector joining the attachment points.

These structural studies on the isolated target domain do not provide any information about potential changes in the labelled target domain after it has been introduced into muscle cells, and until recently, the only way to assess this was by functional assays. The most informative functional assay for TnC in muscle fibres is the calcium control of force production, and maximal calcium-activated force has been measured for each TnC FISS probe used in the studies summarised in this review. Although early studies of this type (e.g. Ferguson et al. 2003) reported a decrease in maximal force of about 20% following the protocol used for the replacement of native by labelled TnC, this was a non-specific effect of the exchange protocol used in that study. Subsequent studies using milder exchange protocols (Sevrieva et al. 2014) showed the maximal force in muscle samples containing each of the labelled TnCs was not significantly different from that in control samples. For every probe used in published FISS studies, the native function of TnC as assessed by this assay was not affected by the mutagenesis, bifunctional rhodamine labelling or TnC exchange. There is a small decrease in the calcium sensitivity of active force for muscle cells containing some of the labelled TnCs, and this has been characterised in detail in the studies using calcium titrations as functional assays (Sun et al. 2006; Sun et al. 2009; Brunello et al. 2023). The rate of isometric force generation and the velocity of unloaded shortening of skeletal muscle fibres into which the labelled TnCs had been exchanged were also similar to those of control fibres (Brunello et al. 2023).

The functional assays described above provide strong but indirect evidence that the structure of TnC and its interactions with the other components of the thin filaments are not altered by mutagenesis or bifunctional rhodamine labelling, but until recently, it was not possible to compare the domain orientations determined by FISS with the results from any other structural method. The present paper reports the first such comparison, using cryo-EM data from isolated cardiac thin filaments, for the orientations of the N- and C-lobes of troponin C. However, this comparison has two major limitations. First, thin filament structure in isolated filaments is likely to be different from that in muscle cells, for the multiple reasons summarised in the “Introduction” section, and, second, the resolution of the cryo-EM data for TnC_N_ and TnC_C_ in isolated thin filaments is currently limited to about 8 Å.

Despite these limitations, the orientations of TnC_C_ and the IT arm of troponin determined by FISS and cryo-EM are almost identical at both low and high [Ca^2+^] (Figs. 6E, F and 7C, D), providing a clear cross-validation of the techniques and extending the cryo-EM result from the isolated filament to the native environment of the muscle cell. In contrast, the orientations of TnC_N_, the calcium-binding regulatory head of troponin, determined by FISS and cryo-EM are clearly different at both low and high [Ca^2+^] (Figs. 6B, C and 7A, B). Both methods detected multiple populations of TnC_N_ molecules with distinct orientations, but the FISS-derived orientations were more widely separated than those determined by cryo-EM.

There are several possible reasons for the different TnC_N_ orientations determined by FISS and cryo-EM. The orientation distribution of TnC_N_ might be genuinely different in muscle cells and in isolated filaments, for example because of thin filament interactions with MyBP-C or the presence of troponin populations with different TnI phosphorylation levels in the muscle cells. Alternatively, one of the methods may not be accurately preserving and reporting the native TnC_N_ orientation distribution, which is likely to reflect a dynamic equilibrium between weak and competing binding sites in the thin filament. In the case of cryo-EM, this effect might be related to the difficulty of preserving such a dynamic distribution in the freezing protocol or to the difficulty of classifying, aligning and averaging images of a small globular domain, accounting for only about 2% of the mass of the repeating unit of the thin filament, in the presence of conformational dispersion at limited spatial resolution. For FISS, either the removal of native side chains by cysteine mutagenesis or the presence of the bifunctional rhodamine probe might disrupt such a weak dynamic equilibrium for TnC_N_. This is not inconsistent with the fact that the method accurately reports the orientation of the IT arm, which is determined by multiple and stable thin filament interactions that are distant from the probe insertion sites.

These uncertainties are likely to be resolved by future studies with the two techniques, which have complementary strengths and limitations. The combination of the two approaches seems particularly powerful. Cryo-EM will produce high-resolution structural information from macromolecular complexes in conditions that are increasingly close to the cellular environment, whilst FISS will extend that information to near-physiological conditions on the physiological timescale to empower mechanistic structure–function studies.

## Implications for the mechanism of muscle regulation by troponin

The improved resolution of troponin structure in both the low- and high-calcium states of isolated thin filaments by cryo-EM represented a major advance in understanding the structural basis of thin filament regulation in muscle. In particular, those studies suggested roles for the C-terminus of TnI and the N-terminus of TnT and for the interaction between the two strands of troponin and tropomyosin in stabilising the off state of the thin filament (Yamada et al. 2020; Risi et al. 2021b, 2024). However, the structure and function of several regions of troponin are unknown, and many questions remain about the structural basis of thin filament regulation (Tobacman 2021). The FISS studies reviewed here provide an independent check on the conclusions of the cryo-EM studies for two domains of troponin in the two well-defined states of low-calcium/relaxation/diastole and high-calcium/active contraction and extend those studies to the native environment of the contraction of both skeletal and heart muscle cells on the physiological timescale.

The cryo-EM studies on isolated thin filaments and FISS studies on heart muscle cells lead to similar conclusions about the orientation of the IT arm of troponin, within the resolution of the methods. The results support the general conclusion that the IT arm acts primarily as a scaffold for the more mobile regions of troponin that have a direct role in calcium-based regulation. At the resolution of the cryo-EM maps and the orientation distributions produced by FISS, the IT arm is relatively insensitive to the binding of calcium to the regulatory sites of TnC_N_ and to the incorporation of thin filaments into the intact lattice of thick and thin filaments. However, the intrinsically high sensitivity of FISS measurements shows that the IT arm does reorient reproducibly by a few degrees on activation of heart muscle and that this signal is due to myosin as well as calcium binding (Sun et al. 2009). A single bifunctional probe on the E-helix of TnC_C_ gives a convenient readout of this motion.

TnC_N_ binds the regulatory calcium ions that trigger muscle activation, two ions in the skeletal muscle isoform and one in the cardiac isoform, and in both cases, the calcium-bound form of TnC_N_ binds the switch peptide of TnI. The cryo-EM maps of the thin filament show that TnC_N_ is close to the filament surface and close to tropomyosin (Yamada et al. 2020; Risi et al. 2021b, 2024). Although the details remain unclear at the currently available resolution, these results suggest that the opening of the TnC_N_ structure following binding of calcium and the TnI switch peptide displaces the C-terminal region of TnI from its inhibitory position on the thin filament, in which it blocks myosin binding directly in addition to the action of tropomyosin in the low-calcium state. The well-defined orientation of TnC_N_ determined by FISS in relaxed skeletal muscle (Fig. 4) suggests that it docks tightly onto the filament surface, and this docking may contribute to the stabilisation of the blocked state, in addition to the more closed structure of TnC_N_, by separating the switch peptide from its TnC_N_ binding site. The different TnC_N_ orientation during active contraction would correspond to an active conformation in which the switch peptide is bound.

The well-defined ‘off’ and ‘on’ orientations of sTnC_N_ in skeletal muscle are not observed in heart muscle, in which cTnC_N_ has multiple conformations in both states. This difference is likely related to the distinct functional requirements of thin filament regulation in heart muscle. The regulation of skeletal muscle is switch-like, and because of the large mass and potential metabolic cost of skeletal muscle in the mammalian body, there would be an evolutionary advantage in stringently reducing the ATP consumption of resting muscles. This is probably also the driver for the so-called super-relaxed state of skeletal muscle (Stewart et al. 2010) and the folded off state of the thick filaments in skeletal muscle (Irving 2017; Craig and Padron 2022). In skeletal muscle, thick and thin filament regulation work in parallel with two positive feedback loops, mechanosensing in the thick filaments and activation of the thin filaments by myosin binding, that together ensure rapid exit from this super-off state (Brunello et al. 2023).

Regulation of heart muscle, in contrast, works like a rheostat rather than a switch, and multiple inputs in addition to the intracellular calcium transient modulate the strength and speed of contraction and relaxation in the heart. The calcium sensitivity of the thin filament is dependent on sarcomere length, binding of the N-terminus of MyBP-C and the phosphorylation levels of MyBP-C, TnI and the myosin RLC, as discussed above. The detailed structural mechanisms responsible for the effects of these additional inputs on the regulatory state of the thin filament are unknown, but the multiple orientations of TnC_N_ may be intermediates on these signalling pathways. For example, the multiple conformations of TnC_N_ in both the off and on states of the cardiac thin filament might correspond to different phosphorylation states. These ideas could be tested in heart muscle cells by future FISS studies in which the additional inputs are controlled.

## Data Availability

No datasets were generated or analysed during the current study.
